# Respiratory-Responsive Vocal Biomarker for Asthma Exacerbation Monitoring: Prospective Cohort Study

**DOI:** 10.2196/68741

**Published:** 2025-09-23

**Authors:** Erik Larsen, Xinyu Song, Dale Joachim, Peter Y Ch'en, Samuel M Green, Emily Hunt, Savneet Kaur, Robin Nag, Olivia Pisani, Sherron Thomas, Victoria Adewunmi, Carlo Lutz, Babak Baghizadeh-Toosi, Jonathan M Feldman, Sunit Jariwala

**Affiliations:** 1 Sonde Health Somerville, MA United States; 2 Albert Einstein College of Medicine Bronx, NY United States; 3 Montefiore Medical Center Bronx, NY United States; 4 Ferkauf Graduate School of Psychology Yeshiva University Bronx, NY United States

**Keywords:** asthma management, respiratory exacerbation, patient engagement, digital health, vocal biomarkers, mobile health, mHealth, remote monitoring

## Abstract

**Background:**

Asthma exacerbations remain a major challenge in asthma management, often due to delayed recognition and limitations of conventional monitoring tools such as peak flow meters and symptom questionnaires. These tools are typically effort dependent or retrospective, making them less suited for continuous, real-time monitoring. A novel, smartphone-based respiratory-responsive vocal biomarker (RRVB) may offer an accessible and noninvasive approach for dynamic assessment of respiratory health. This RRVB has previously demonstrated generalizable performance in cross-sectional cohorts across multiple respiratory conditions, including asthma, chronic obstructive pulmonary disease, and COVID-19, in populations spanning India and the United States. This study extended this work by evaluating the real-world, longitudinal performance of the same RRVB tool for daily asthma exacerbation monitoring via smartphones in home settings.

**Objective:**

This study aimed to evaluate the efficacy of the RRVB as a convenient real-time tool for monitoring asthma exacerbations and respiratory states in a real-world, longitudinal setting.

**Methods:**

In this prospective cohort study, 84 adult patients with asthma were enrolled from an academic medical center and followed for 90 days. Participants submitted daily 6-second voice samples and conducted peak expiratory flow measurements and surveys, including symptom reports and asthma control assessments. RRVB scores were generated in real time on the app. Asthma states (normal function, mild event, and exacerbation) were defined based on both peak expiratory flow and self-reported well-being. Risk ratios were calculated to assess the predictive validity of RRVB scores for identifying exacerbation events. Engagement was measured via frequency of completed sessions, and participant experience was evaluated through exit surveys.

**Results:**

RRVB scores significantly stratified asthma states. The risk of experiencing an exacerbation was 2.15 times higher (95% CI 1.62-2.85; *P*<.001) with elevated RRVB scores and 3.57 times higher (95% CI 2.70-4.73; *P*<.001) using normalized scores adjusted for individual characteristics. RRVB scores did not significantly correlate with the Asthma Control Test (risk ratio=1.17, 95% CI 0.96-1.44; *P*=.12), highlighting its role as a momentary signal rather than a proxy for longitudinal control. Engagement was moderate or higher (≥26 total app sessions) in 58% (49/84) of participants. Among survey respondents, 93% (43/46) found the app easy to use, 89% (41/46) reported a positive overall experience, and 87% (40/46) indicated that they would use a similar tool in the future. Fewer participants (32/46, 70%) reported understanding the RRVB scores, suggesting a need for improved score interpretability and user guidance in future implementations.

**Conclusions:**

The RRVB tool demonstrated effective real-time detection of asthma exacerbations and dynamic respiratory states, supporting its potential as a noninvasive, user-friendly, and physiologically grounded digital biomarker for asthma monitoring. These findings provide foundational evidence for broader deployment and integration of voice-based tools to support proactive, real-world asthma management.

**Trial Registration:**

ClinicalTrials.gov NCT05850390; https://clinicaltrials.gov/study/NCT05850390

## Introduction

### Background

Current asthma management strategies rely heavily on regular symptom and lung function monitoring to prevent exacerbations and optimize treatment [1]. Traditionally, spirometry and peak flow meter readings have been used to assess lung function; however, these require clinical visits or patient-operated devices (eg, peak flow meters), which may not be readily accessible or optimally used [2]. Furthermore, these methods provide only episodic snapshots of lung function, potentially missing the subtle day-to-day fluctuations that can precede an exacerbation. Research indicates that regular monitoring combined with asthma education are of primary importance in asthma management [3].

Advancements in digital health have opened up avenues for more continuous and patient-centered monitoring approaches. Among digital technologies, vocal biomarkers have emerged as noninvasive, accessible solutions [4-6] leveraging changes in voice characteristics that can reflect underlying pathophysiological changes, including respiratory conditions. With the ubiquity of smartphones and other voice-enabled devices, vocal biomarkers can be captured and analyzed for real-time feedback on respiratory status. For asthma applications, such research has focused on analysis of cough, breath, wheeze sounds [7-11], and speech [12-14], attempting to identify or characterize asthma with high sensitivity and specificity. However, these studies have historically faced limitations such as small sample sizes, brief study durations, retrospective or laboratory-based testing, and limited real-world validation of automated scoring systems [11]. Our study design advances the field in several of these areas (duration, use of a predesign and automated scoring algorithm, ecological validity, and transparent index test comparators).

### Objectives

This study aimed to address these shortcomings by building on a previously developed and validated respiratory-responsive vocal biomarker (RRVB) that operates on user devices in real-world settings [15]. The RRVB tool analyzes 6-second held vowel elicitations measured on smartphones to extract and interpret vocal features correlating with respiratory function. The RRVB has been shown to provide approximately 70% sensitivity and specificity in distinguishing diagnoses of asthma, chronic obstructive pulmonary disease (COPD), interstitial lung disease, persistent cough, and COVID-19 from healthy controls [15]. This performance was demonstrated in 2 independent studies from India and the United States including English and 5 Indian languages.

Our study linked the need for improved asthma management strategies with the potential of the RRVB to provide real-time respiratory function assessments. It piloted the RRVB’s utility as a reliable asthma monitoring tool by correlating RRVB scores with exacerbations. In addition, this study explored participant engagement with the RRVB tool and collected feedback on its usability and perceived benefits, thereby contributing to a broader understanding of vocal biomarker tools in asthma management. The hypothesis was that the RRVB scores could accurately differentiate between exacerbations and normal respiratory states in patients with asthma as defined by combined peak flow and symptom measures and that participants would report favorably on its potential as an asthma management tool. We combined peak flow (objective) and symptom (subjective) measures to ensure a robust comparison of RRVB scores to asthma state, reflecting the common clinical practice of assessing asthma holistically and recognizing that neither peak flow nor symptoms alone fully capture a patient’s condition.

## Methods

### Study App and RRVB

**Figure 1:**
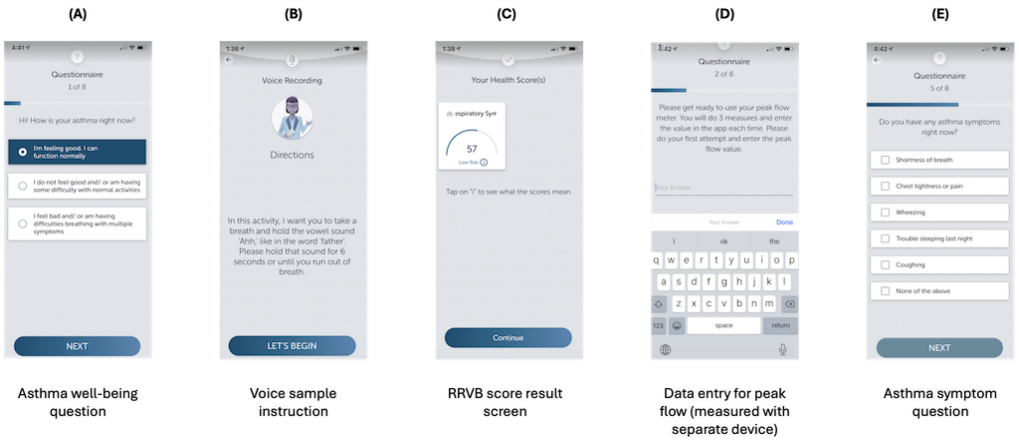
Selected screenshots (not exhaustive) illustrating study app user experience: (A) asthma well-being question (multiple choice), (B) voice sample instruction, (C) respiratory-responsive vocal biomarker (RRVB) score result, (D) peak flow result entry, (E) asthma symptoms question (multiple select). An app study session always flows through the same set of activities, ensuring consistent data collection.

The study app was downloaded and used on participant smartphones to collect voice samples and questionnaire responses and was compatible with iOS and Android devices. Selected screens illustrating the app user interface and flow are shown in Figure 1. Voice samples (6-second held [ɑ] vowels, as in *father*) were processed to generate RRVB scores, which were displayed to the user on a result screen in real time (5-10–second latency). Peak flow and subjective asthma reports were collected through questionnaires on the app. Participants were encouraged to use the app as part of their daily routines, with push notifications reminding them to use it. A study session was defined as the successful completion of 1 full interaction with the study app, including both voice samples and questionnaire responses. If the session was not completed (ie, if the participant exited the app before finishing the voice or questionnaire components), it was treated as incomplete and not recorded in the study database. Incomplete study sessions were not penalized, but persistent low completion rates could lead to participant withdrawal, as explained in the Study Site and Recruitment section.

Development and validation of the RRVB on asthma and other respiratory conditions included various cohorts in multiple geographies [15]. The RRVB model generated a composite score from 0 to 100 indicating the relative risk of respiratory symptoms. Using a threshold score of 65, the model differentiated patients with respiratory symptoms from healthy controls with approximately 70% sensitivity and specificity. In addition, 2 quality control algorithms operated on voice samples and score results to ensure robust and valid outcomes (described in the Elicitation Check and 2-Sample Scoring section).

### Study Design

#### Study Site and Recruitment

Participants were recruited during routine clinical visits at Montefiore Medical Center (Bronx, New York, United States) via the Allergy and Immunology clinic (both in person and remote) and the emergency department. Interested participants installed the study app on their smartphones. During enrollment, either in person or via telehealth, research assistants provided one-time training on app use. This included a step-by-step walk-through of the full study session flow (voice recording, questionnaire completion, and peak flow meter technique), with the participant completing 1 session under supervision to ensure comfort and accuracy. No additional structured training was provided after this initial session. Participants could contact study staff with questions; however, app-related inquiries were minimal.

This study aimed to recruit 70 participants, with provisions to recruit more to account for attrition, defined as completing <2 sessions per week over 3 weeks without foreseeable improvement despite re-engagement efforts. Participants observed the RRVB scores on the study app but were instructed not to use them for asthma management.

#### Eligibility Criteria

Patients at the study site were eligible if they had a diagnosis of persistent asthma confirmed via chart review or allergy with asthma as a comorbidity, were prescribed a controller medication, were aged ≥18 years, and could read and speak English. COPD was allowable as a comorbidity. Participants needed to own a smartphone and be willing to use the study app. Exclusions included speech disorders, difficulties using smartphones, end-stage COPD or other conditions requiring home oxygen, anticipated difficulties with daily peak flow meter readings, or participation in other asthma-focused medication studies.

#### Study Schedule and Incentives

**Figure 2:**
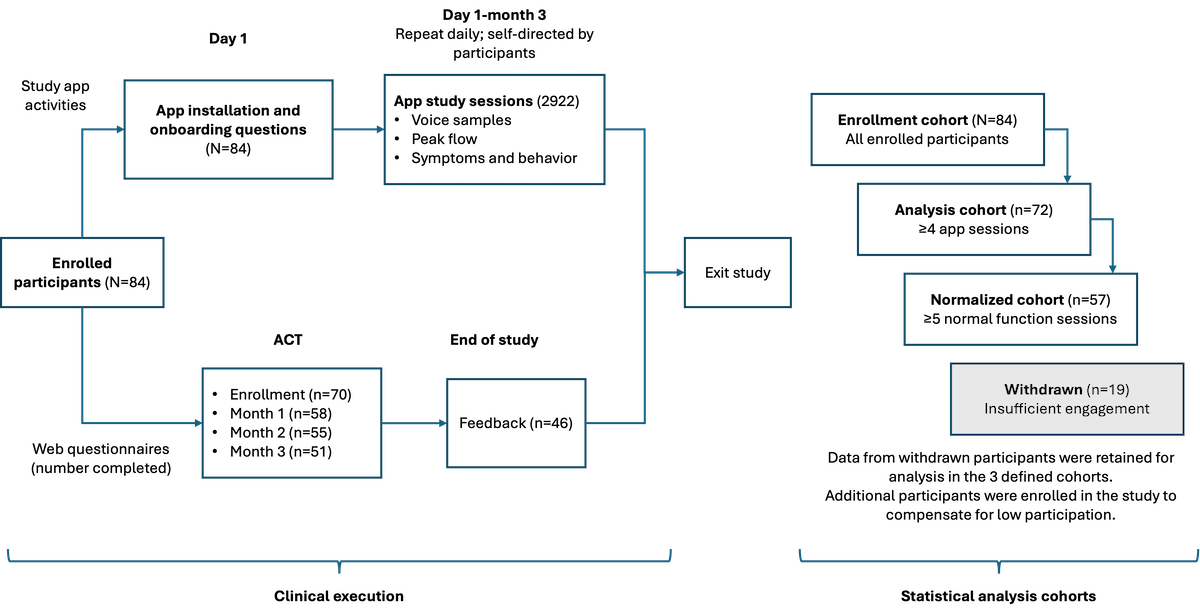
Overview of participant flow and study cohorts. Respiratory-responsive vocal biomarker (RRVB) sessions are conducted via the study app, whereas the Asthma Control Test (ACT) and study feedback are obtained online. Numbers indicate participants by cohort and completion of app sessions and questionnaires, as explained in the main text.

The study flow is illustrated in Figure 2. The 3-month study aimed to capture sufficient voice samples for normal and exacerbation states and gather participant feedback on the RRVB tool.

#### Assessments and Data Collection

Enrollment data were collected via the study app, including demographic, medical, and behavior-related questions. Each participant was provided with a single electronic peak flow meter (Microlife PF 100) to record peak expiratory flow (PEF) values entered into the app. The highest of 3 values per session was used for analysis, categorized into green, yellow, and red zones based on personal best percentages. Personal best PEF was calculated from data collected during the study (see the Statistical Methods section). Asthma Control Test (ACT) scores were collected using a web-based survey platform (SurveyLex) [16] online at enrollment and monthly, categorized into well controlled (20-25), not well controlled (16-19), and poorly controlled (5-15) asthma.

The study app captured patient-reported outcomes and asthma management practices during each session after voice sample collection, including the following:

1. Asthma well-being; participants answered the following question—“How is your asthma right now?”—with the options “I’m feeling good. I can function normally,” “I do not feel good and/or am having some difficulty with normal activities,” and “I feel bad and/or am having difficulties breathing with multiple symptoms.” This, along with peak flow, determined respiratory state (normal function, mild event, or exacerbation).
2. Asthma symptoms; participants answered the following question—“Do you have any asthma symptoms right now?”—with the following options for multiple selection: “Shortness of breath,” “Chest tightness or pain,” “Wheezing,” “Trouble sleeping last night,” “Coughing,” and “None of the above.”
3. Triggers; participants answered the following question—“If you are having asthma symptoms right now, are you aware of any triggers”—with the following options for multiple selection: “Tobacco smoke,” “Air pollution,” “Pets,” “Exercise,” “Disinfectant,” “Other allergies,” “Stress,” “Other reason (not listed),” “I don’t know the trigger,” and “Not applicable (no symptoms).”
4. Symptom severity; participants answered the following question—“How would you rate your asthma symptoms right now?”—with the options “Severe,” “Moderate,” “Mild,” and “Not applicable (no symptoms).”
5. Medication use; participants answered the following question—“Have you used your rescue inhaler?”—with the options “No, haven’t used it today,” “No, but I plan to use it after finishing this app session,” “Yes, I used it earlier today to relieve symptoms,” and “Yes, I used it earlier today prior to exercise.”

At the study’s end, feedback on the RRVB tool was gathered via an online survey including statements about the app’s usability and participant interaction rated on a 5-point Likert scale from *completely disagree* to *completely agree*. Participants rated their affinity for using the app, ease of use, clarity of understanding results, perceived helpfulness in managing asthma, preference for vocal biomarker analysis over traditional peak flow measurement, and interest in continuing app use after the study. The survey also included open-ended questions about the most and least favorable aspects of the app and suggestions for improvement.

#### End Points

##### Categorization of Respiratory State

Data from each session categorized participants’ respiratory state into normal function, mild events, and exacerbations. Exacerbations were defined as (1) PEF reduction of >20% from their personal best (yellow or red zone) and (2) reporting not feeling good or feeling bad in response to the following question: “How is your asthma right now?”

This definition aligns with established clinical practice [17], which typically integrates both objective and subjective data to assess asthma state. As neither PEF nor symptoms alone are fully reliable in capturing the complexity of a patient’s respiratory state, we combined both measures to provide a more comprehensive and potentially more accurate label. Mild events were defined as sessions meeting either the PEF or asthma well-being criterion but not both. Normal function were defined as sessions meeting neither criterion.

##### Risk Ratio for Respiratory States

The primary end point was the risk ratio (RR) for exacerbations comparing 2 RRVB score ranges: low risk (0-64) and high risk (65-100). The RR was calculated as the ratio of the prevalence of exacerbations (exacerbation count divided by exacerbation+normal function count) within the high-risk versus the low-risk range. The RRVB score threshold of 65 was based on previous benchmarks [15]. RR was also calculated for mild events and asthma control using the same approach and RRVB score ranges. RR estimates are reported with the corresponding 95% CIs and 2-sided *P* values derived from SE estimates to assess whether the RR significantly differed from the null hypothesis value of 1.0.

A normalized analysis accounted for individual variability in normal function. This adjusted analysis aimed for an improved RR by reducing variability unrelated to respiratory function by calculating a reference RRVB score for each participant representing normal function, as described in the Statistical Methods section. A normalized RRVB score threshold to separate high-risk versus low-risk ranges was calculated post hoc as the 70th percentile of the normalized RRVB reference score distribution.

##### Engagement and Feedback

Secondary end points included engagement levels and retention in month 3. Engagement level groups were defined by total app sessions: high (≥52), medium (26-51), and low (1-25). Engagement was measured using the number and percentage of participants in each group and the average number of sessions per month. Retention was calculated in 2 ways: the proportion of participants with at least one session in month 3 (minimum use) and those with at least 8 sessions in month 3 (consistent use). Participant feedback, including multiple-choice questions on usability and helpfulness and free-text responses, was summarized by theme using a pragmatic approach. Themes were initially identified using a language model (GPT-4 Turbo; OpenAI) and then edited for accuracy and completeness by the study authors against the raw responses. Due to the brevity of the comments, no formal qualitative coding method was applied.

### Ethical Considerations

The study design followed Standards for Reporting of Diagnostic Accuracy Studies guidelines [18] and was approved by the Institutional Review Board of the Albert Einstein College of Medicine (078142). It was retrospectively registered on ClinicalTrials.gov (NCT05850390) on April 28, 2023, after study initiation (December 14, 2021) but before completion of participant enrollment. Participants were informed about incentives, including US $25 for completing monthly ACT questionnaires and US $15 weekly if conducting ≥4 study app sessions, and that these were not contingent on specific outcomes. Study data were deidentified through the use of numeric participant identifiers that were used for all data collection and analysis. App data were stored in a secure cloud repository hosted on Amazon Web Services. Informed consent was provided electronically through the study app during the onboarding and training session in the presence of the research associate, who was available to answer study-related questions.

### Cohort Definitions

As illustrated in Figure 2, the enrollment cohort included all participants who began the study and was used for analyzing engagement and retention. The analysis cohort, defined as participants with at least 4 study sessions, was used to analyze correlations among the RRVB, respiratory state, and ACT. The normalized cohort, a subset of participants in the analysis cohort with at least 5 normal function sessions, enabled estimation of reference RRVB and normalized scores.

### Statistical Methods

#### Sample Size Determination

Sample size was estimated assuming approximately 70% sensitivity and specificity for RRVB score classification of respiratory state, consistent with performance of RRVB in cross-sectional screening [15]. With a Bonferroni-corrected α of .0125 (for 4 planned comparisons: exacerbation vs normal and mild vs normal for both raw and normalized RRVB scores) and 80% power, a minimum of 37 participants was required. To accommodate 25% anticipated missed study sessions during exacerbation days and 25% of participants without exacerbation-like events during the study, the recruitment goal was increased to 70 participants (rounded up), with flexibility to enroll additional participants as needed to compensate for attrition.

#### Elicitation Check and 2-Sample Scoring

An elicitation check algorithm ensured voice sample quality and reliability, rejecting those not meeting specific acoustic criteria. This real-time algorithm prompted participants to rerecord samples if necessary up to 3 times. To further enhance RRVB score robustness and reduce measurement variability, a 2-sample scoring algorithm was applied to scores post hoc. Two voice samples were obtained in each study app session, and the algorithm accepted scores only if their difference was within 10 points, averaging them to provide 1 final score. Sessions with score differences exceeding this threshold were deemed implausible and excluded from analysis.

#### Relative PEF

PEF values were expressed as a percentage of the participant’s personal best derived from study data as follows: first, PEF values of >1000 L per minute were removed as errors, and the highest remaining valid measurement per session was used. Personal best was calculated as the highest value not exceeding the 95th percentile of reported PEF values. PEF readings of <20% or >120% of this personal best were excluded as clinically implausible. PEF values were categorized into green (≥80%), yellow (50%-79%), and red (<50%) zones for analysis.

#### Reference RRVB and Score Normalization

For each participant, a reference RRVB score was calculated as the median score from all sessions labeled as normal function. These sessions were defined as previously described (both PEF and symptom reports indicating no significant respiratory impairment), reflecting the participant’s unimpaired respiratory function. As there was no predefined baseline period, this reference RRVB score estimate was derived from all applicable sessions spanning the entire study period. To assess changes in respiratory status, we normalized the RRVB scores by subtracting this reference value from the raw RRVB scores, providing a measure of deviation from the participant’s usual respiratory state.

#### RRVB Change Score Analysis

To determine RRVB score changes between respiratory state transitions, an individual change score analysis was conducted. Respiratory state changes were categorized based on individual participants between successive sessions, and RRVB score changes were grouped according to each type of change. Change score distributions were described using mean and SE.

#### Time Averaging of RRVB for ACT Analysis

To compare ACT results with RRVB scores, we averaged RRVB scores within a 4-week window centered on the ACT date, extending to 2 weeks before and after. This approach was chosen to ensure consistent linkage of ACT and RRVB data throughout the study period, especially for the enrollment ACT, where no previous RRVB data existed. While the ACT reflects a 4-week retrospective assessment, asthma control generally changes gradually, so control in the 2 weeks following the ACT date is expected to correlate closely with that in the 2 weeks before. By centering the window, we were able to capture relevant RRVB data for all ACT assessments, including those at enrollment and at the end of the study, maintaining a consistent method for comparing asthma control and vocal biomarker data.

## Results

### Enrollment and Cohorts

This study enrolled 84 participants (n=68, 81% in person and n=16, 19% remotely) from December 2021 to August 2023, forming the enrollment cohort (Figure 2). Of these 84 participants, 12 (14%) completed <4 sessions, leaving 72 (86%) in the analysis cohort. Of these 72 participants, 15 (21%) had <5 normal function sessions, resulting in 57 (79%) participants in the normalized cohort. The enrollment cohort included 23% (19/84) of participants who were withdrawn due to lack of engagement (data were retained for analysis), and additional participants were enrolled to increase data availability.

### Participant Characteristics

#### Demographics and Comorbidities

The demographic and clinical characteristics of the enrollment cohort are shown in Table 1. The enrollment cohort was predominantly female (68/84, 81%) with a mean age of 39.8 (SD 11.6) years. Most (49/84, 58%) identified as Hispanic or Latino, and the cohort had a diverse racial composition: 44% (37/84) Black or African American individuals, 15% (13/84) White individuals, and 42% (35/84) reporting “other.” On the basis of self-reported data, 95% (80/84) of the participants had one or more comorbid condition—the most common were seasonal allergies (61/84, 73%) and other allergies (51/84, 61%), followed by hypertension and sinus conditions (24/84, 29% each) and obesity (23/84, 27%). Mental health conditions were prevalent (39/84, 46%). Most participants (53/84, 63%) used iPhones, followed by Samsung smartphones (26/84, 31%).

**Table 1:** Sociodemographic and health data reported by participants at study onboarding (N=84).

Variable			Participants, n (%)
**Sex**			
	Female	68 (81)	
	Male	16 (19)	
**Age (y)**			
	18-30	23 (27)	
	31-45	30 (36)	
	≥46	31 (37)	
**Ethnicity—Hispanic or Latino**			
	Yes	49 (58)	
	No	35 (42)	
**Race^a^**			
	American Indian or Alaska Native	3 (4)	
	Asian	2 (2)	
	Black or African American	37 (44)	
	Native Hawaiian or other Pacific Islander	0 (0)	
	White	13 (15)	
	Other	35 (42)	
**Highest degree or level of education**			
	Some high school	8 (10)	
	High school	33 (39)	
	Associate’s degree	12 (14)	
	Bachelor’s degree	24 (29)	
	Master’s degree	5 (6)	
	Professional or doctorate degree	0 (0)	
	Trade school	2 (2)	
**Approximate household income (US $)**			
	<20,000	32 (38)	
	20,001-40,000	20 (24)	
	40,001-60,000	14 (17)	
	60,001-80,000	9 (11)	
	80,001-100,000	4 (5)	
	≥100,000	5 (6)	
**Health insurance coverage**			
	No health insurance	0 (0)	
	Private health insurance	26 (31)	
	Medicaid, Medicare, or government medical assistance	55 (65)	
	Some other insurance	3 (4)	
**Other health conditions^a^**			
	Seasonal allergy	61 (73)	
	Allergy (other type)	51 (61)	
	Hypertension	24 (29)	
	Sinus condition	24 (29)	
	Obesity	23 (27)	
	Diabetes	16 (19)	
	Sleep apnea	16 (19)	
	Reflux disease	15 (18)	
	Skin condition	15 (18)	
	Chronic obstructive pulmonary disease	4 (5)	
	Chronic heart failure	0 (0)	
**Mental health conditions^a^**			
	Depression	24 (29)	
	Anxiety	32 (38)	
	Other mental health condition	9 (11)	
	None of the above	45 (54)	
	More than one mental health condition	21 (25)	
**Smartphone brand**			
	iPhone	53 (63)	
	Samsung	26 (31)	
	Other	5 (6)	

^a^More than one category may apply, so the percentages may add up to >100%.

#### Asthma Characteristics

Asthma severity, determined from health records based on physician diagnosis following clinical guidelines [19], is reported in Table 2. A total of 43% (36/84) of the participants had mild asthma (5/36, 14% intermittent and 31/36, 86% persistent), 33% (28/84) had moderate persistent asthma, and 24% (20/84) had severe persistent asthma. Average ACT scores, also shown in Table 2, indicated that more than half (42/74, 57%) of the participants had poor asthma control (not including participants without ACT data). These findings aligned with the study’s objective to include participants likely to experience exacerbations. Prevalence of poor asthma control increased with asthma severity.

**Table 2:** Asthma control (Asthma Control Test [ACT]; participant reported) and asthma severity (clinician diagnosis; health records).

Level of asthma control from participant ACT responses averaged across multiple ACTs^a^	Enrollment cohort (n=84), n (%)	Mild asthma (intermittent and persistent; n=36), n (%)	Moderate persistent asthma (n=28), n (%)	Severe persistent asthma (n=20), n (%)
Well controlled (ACT score≥20)	16 (22)	11 (32)	3 (13)	2 (12)
Not well controlled (ACT score=16-19)	16 (22)	9 (26)	6 (26)	1 (6)
Poorly controlled (ACT score≤15)	42 (57)	14 (41)	14 (61)	14 (82)
No ACT responses	10 (12)	2 (6)	5 (18)	3 (15)

^a^Participants without ACT data were not included in percentage calculations.

#### Engagement and Retention

Participants engaged with the study app an average of 34.8 (26.0) times over 3 months, amounting to 2922 total study app sessions. Engagement and retention statistics for the enrollment cohort and split by engagement subgroup are provided in Table 3. The high-engagement group (28/84, 33% of participants) conducted approximately 22 sessions per month (5 per week) with minimal drop-off and had 100% (28/28) consistent use in month 3. The medium-engagement group (21/84, 25% of participants) started at approximately 16 sessions per month (every other day), but use dropped 50% (approximately 8 sessions per month) by month 3, with 86% (18/21) using the app at least once and 48% (10/21) using it consistently (≥8 uses). The low-engagement group (35/84, 42% of participants) started with approximately 6 uses per month (1.5 per week) and saw a 79% (approximately 1 session per month) drop-off by month 3. Only 31% (11/35) had any use in month 3, and only 3% (1/35) used the app consistently.

**Table 3:** Participant engagement measured as average monthly sessions and retention in month 3. App use varied considerably between participants with a high (≥52), medium (26-51), and low (1-25) number of total app sessions. Retention in month 3 is calculated as the proportion of participants with any engagement (≥1 session) and sustained engagement (≥8 sessions; N=84).

Engagement group	Participants, n (%)	Sessions per month, mean (SD)					Month 3 retention, n (%)	
		Month 1	Month 2	Month 3	Total	≥1 session		≥8 sessions
All	84 (100)	14.0 (9.3)	11.2 (9.8)	9.6 (9.9)	34.8 (26.0)	57 (68)		39 (46)
High	28 (33)	22.1 (8.4)	22.0 (6.6)	21.4 (6.1)	65.5 (13.7)	28 (100)		28 (100)
Medium	21 (25)	16.2 (5.3)	12.1 (3.8)	7.7 (5.4)	36.0 (5.8)	18 (86)		10 (48)
Low	35 (42)	6.2 (4.0)	2.1 (2.8)	1.3 (2.7)	9.5 (6.8)	11 (31)		1 (3)

### Exacerbation Monitoring

**Figure 3:**
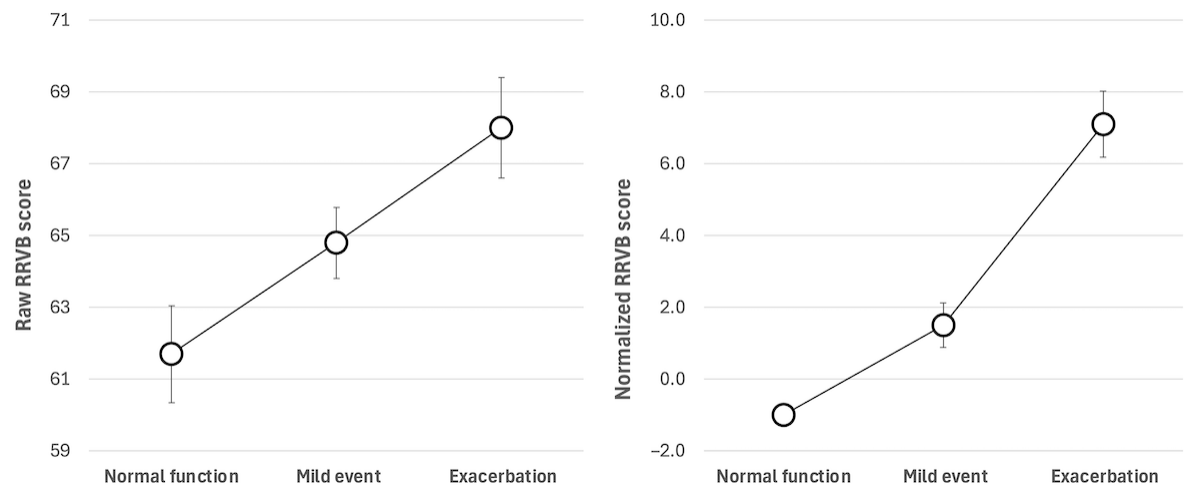
Respiratory-responsive vocal biomarker (RRVB scores) (mean and SEM) for different respiratory states, averaged across participants and sessions: raw scores (left) vs normalized scores (right, subtracting individual baselines). Respiratory state was defined as baseline (“feeling good”, normal peak expiratory flow [PEF]), mild events (not “feeling good” or reduced PEF), and exacerbations (not “feeling good” and reduced PEF).

Figure 3 (left) shows mean RRVB scores across respiratory states, from normal function (*feeling good* and normal PEF; mean score ±S.E. 61.7±1.4) to mild events (either not *feeling good* or reduced PEF; mean score 64.8±0.99) to exacerbations (not *feeling good* and reduced PEF; mean score 68.0±1.4). As expected, the scores increased from normal function to exacerbation by 6.3 points. This pattern was also present for normalized RRVB scores, as indicated in Figure 3 (right), with more pronounced changes—mean normalized scores increased from −1.0±0.31 at normal function to 1.5±0.62 for mild events and 7.1±0.92 for exacerbations, for a total increase of 8.1 points. Mean reference RRVB scores varied minimally (<2.5 points) as a function of level of asthma control (data not shown).

The RRVB scores’ ability to differentiate respiratory states is shown in Table 4, indicating the RR for exacerbations as 2.15 (95% CI 1.62-2.85; *P*<.001). For mild events, the RR was 1.42 (95% CI 1.23-1.65; *P*<.001). These results confirm the effectiveness of RRVB scores in distinguishing respiratory states, especially for exacerbations.

Following normalization, RRVB scores were segmented into high- and low-risk categories using the calculated threshold of 3.7. Table 4 shows that the RR for exacerbations was 3.57 (95% CI 2.70-4.73; *P*<.001). For mild events, the RR was 1.31 (95% CI 1.13-1.53; *P*<.001). Normalization significantly improved risk stratification for exacerbations by reducing score variability unrelated to respiratory function. Normalization did not improve risk stratification for mild events.

Table 5 shows the RRVB score changes for respiratory state transition assessed within individuals. Most transitions involved participants remaining in the same state, whereas direct transitions from normal function to exacerbation or vice versa were uncommon. The data reveal that RRVB scores remained stable when respiratory states did not change. When transitions occurred, the magnitude of RRVB score change varied—approximately 3.5 points for shifts between normal function and mild events and 2 to 3 points for changes between mild events and exacerbations. Shifts between normal function and exacerbations resulted in an RRVB score change of approximately 6 points, suggesting a cumulative effect of worsening respiratory conditions on RRVB scores.

**Table 4:** Risk ratios (RRs) for exacerbations (not feeling good and reduced peak flow), calculated as the ratio of prevalence (exacerbations/exacerbations+normal function) in high versus low respiratory-responsive vocal biomarker (RRVB) score ranges and analogously for mild events (not feeling good or reduced peak flow but not both). Results correspond to raw and normalized RRVB scores.

Event type		High-risk RRVB score range (raw score≥65, normalized score≥3.7) session count	Low-risk RRVB score range (raw score<65, normalized score<3.7) session count	RR for high vs low RRVB score range (95% CI)	*P* value
**Raw RRVB score-analysis cohort**					
	Exacerbation	167	57	2.15 (1.62-2.85)	<.001
	Mild	390	194	1.42 (1.23-1.65)	<.001
	Normal function (reference)	704	581	1.00	—^a^
**Normalized RRVB score-normalized cohort**					
	Exacerbation	124	65	3.57 (2.70-4.73)	<.001
	Mild	186	293	1.31 (1.13-1.53)	<.001
	Normal function (reference)	382	883	1.00	—^a^

^a^Not applicable.

**Table 5:** Respiratory-responsive vocal biomarker (RRVB) score changes between respiratory states within consecutive study sessions of the same participant averaging all data from the analysis cohort. Each transition type is characterized using the mean and SE of the RRVB score change and count of transition types.

Initial state	Final state—score change, mean (SE)		
	Normal function	Mild event	Exacerbation
Normal function	0.1 (0.4)	3.5 (1.0)	6.3 (1.8)
Mild event	-3.4 (1.0)	0.0 (0.8)	1.9 (1.6)
Exacerbation	-5.4 (2.4)	-3.1 (1.6)	0.3 (1.1)

### Asthma Control

**Figure 4:**
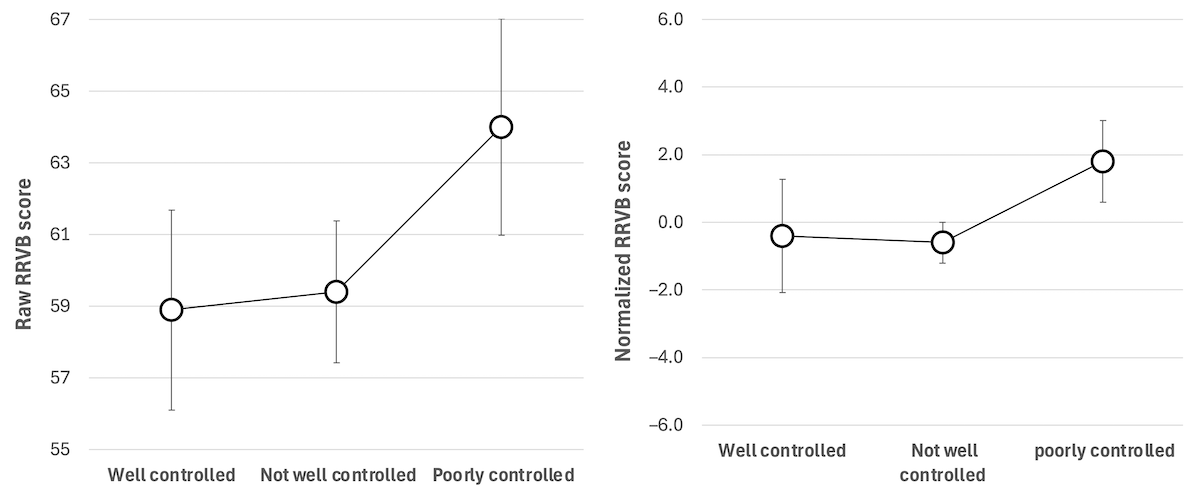
Time-averaged respiratory-responsive vocal biomarker (RRVB) scores (mean and SEM) for varying asthma control levels, averaged across participants and sessions. Well controlled: Asthma Control Test (ACT) score ≥20, not well controlled: ACT score=16-19, poorly controlled: ACT score ≤15.

Of the 234 ACT assessments conducted during the study, 201 (85.9%) were eligible for correlation analysis with RRVB scores based on the availability of at least one RRVB result within 2 weeks before or after the ACT assessment date. As expected, RRVB scores increased as asthma control (ACT) decreased (Figure 4). RRVB score increase as a function of asthma control was less pronounced compared to RRVB score increases as a function of momentary respiratory state, in particular for normalized RRVB scores, which only increased by approximately 2 points from well-controlled to poorly controlled asthma. Thus, normalization may obscure underlying differences in asthma control apparent in unadjusted scores. RR estimates for poorly controlled or not well controlled asthma were 1.17 and 1.15, respectively, with CIs that included 1.0 (Table 6). This suggests insufficient statistical evidence to support the effectiveness of time-averaged RRVB scores in stratifying levels of asthma control.

**Table 6:** Risk ratios (RRs) for poor asthma control calculated as the ratio of prevalence (poor/poor+well controlled) in high versus low respiratory-responsive vocal biomarker (RRVB) ranges and analogously for not well-controlled asthma. The results shown correspond to raw and normalized RRVB scores. The high- and low-risk categories are applied to time-averaged RRVB scores.

Level of asthma control			High-risk RRVB score range (raw score≥65, normalized score≥3.7) session count		Low-risk RRVB score range (raw score<65, normalized score<3.7) session count		RR for high vs low RRVB score range (95% CI)		*P* value
**Raw RRVB score-analysis cohort**									
	Poor (ACT^a^ score<16)	61		50		1.17 (0.96-1.44)		.12	
	Not well controlled (ACT score=16-19)	22		24		1.15 (0.77-1.71)		.51	
	Well controlled (ACT score≥20; reference)	18		26		1.00		—^b^	
**Normalized RRVB score-normalized cohort**									
	Poor (ACT^a^ score<16)	30		61		1.10 (0.87-1.40)		.41	
	Not well controlled (ACT score=16-19)	6		38		0.64 (0.33-1.26)		.20	
	Well controlled (ACT score≥20; reference)	11		31		1.00		—^b^	

^a^ACT: Asthma Control Test.

^b^Not applicable.

### Subgroup Analysis

Engagement and RRVB performance across demographic and clinical subgroups illustrate how the RRVB’s predictive accuracy varied within the cohort (Table S1 in Multimedia Appendix 1). The prevalence of exacerbations highlights differences in asthma characteristics within these subgroups.

Engagement differences were analyzed by comparing mean app session estimates and identifying significant effects through nonoverlapping CIs. Substantial effects were found for gender and age range. Lower engagement was observed in male individuals and participants aged <30 years, indicating challenges in consistently engaging these individuals over time. Ethnicity did not significantly affect engagement, indicating good potential to serve diverse populations. Lower engagement approached significance in Black or African American participants. Asthma disease severity (medical record) and asthma control (ACT) were not significantly associated with engagement.

An increasing number of comorbidities was positively correlated with engagement, particularly among participants with ≥4 diagnosed conditions, who engaged at significantly higher rates than those with 0 to 1 or 2 to 3 comorbidities. This trend was also observed for mental health diagnoses, where participants with conditions such as depression or anxiety demonstrated significantly higher engagement than those without these conditions. When analyzing specific comorbidities (not included in Table S1 in Multimedia Appendix 1), 7 out of 10 conditions surveyed were associated with significantly higher engagement. This may suggest that individuals with multiple or complex health conditions may be more motivated to engage with digital health tools, possibly due to a greater perceived need for consistent asthma monitoring.

Exacerbation detection performance, calculated via RRs and comparing raw versus normalized RRVB score analysis, provided several insights. Normalized RRVB scores reduced subgroup differences in RRs, eliminating the statistically significant differences for gender and age observed in the raw RRVB score analysis. This suggests that certain subgroup factors may correlate with RRVB scores independently of respiratory function. Normalized RRVB scores improved RR estimates in 17 of 24 subgroup variables, and where it did not, the CIs overlapped. Nearly all subgroups yielded statistically significant RRs in the normalized analysis except in cases in which low exacerbation prevalence led to wide CIs.

### Participant Feedback

#### Overview

Feedback (Table S2 in Multimedia Appendix 1) was provided by 55% (46/84) of the participants in the enrollment cohort. Overall, the survey responses reflected a strong endorsement of the RRVB-based smartphone tool. A large majority of participants agreed (somewhat or completely) that the app provided a positive user experience (41/46, 89%) and was easy to use (43/46 93%) and that they would be willing to use a similar tool in the future (40/46, 87%). Agreement was slightly lower regarding participants’ understanding of the RRVB scores (32/46, 70%), likely due to limited guidance on the app, and the perception that it offered a better method for asthma assessment than peak flow meters (33/46, 72%). Nonetheless, 78% (36/46) found the RRVB scores helpful for self-assessment of their asthma status, supporting the tool’s potential value for self-monitoring.

#### Best Thing About the App

Participants were asked the following: “The best thing about checking my asthma by recording my voice on my smartphone is...” Positive feedback focused on the tool’s convenience, ease of use, and ability to monitor asthma regularly without leaving home. Participants valued the portability and personalized nature of the app, which fostered a sense of control and active management. Some noted the correlation between sustaining a sound and using a peak flow meter, likening it to a gamified aspect of health monitoring.

#### Worst Thing About the App

Participants were asked the following: “The worst thing about checking my asthma by recording my voice on my smartphone is...” Responses highlighted technical and practical challenges. Technical issues included difficulties with the app not opening or recognizing recordings, requiring repetition. The need for quiet during recordings posed a challenge for those with children or background noise. Some participants were concerned about the accuracy of readings and whether the app’s feedback was sufficiently instructive, particularly regarding when medical intervention might be necessary. The app’s occasional failure to save sessions and the daily routine of using the app also contributed to user burden.

#### Making the App More Useful

Participants were asked the following: “One thing that would make an app like this more useful for people with asthma is...” A common suggestion was implementing reminders for regular use or medication. Push notifications were mentioned, suggesting that some participants did not notice or disallowed these during app setup. Participants also wanted clearer explanations of scores and their health implications, including when to use a rescue inhaler or seek emergency care. Although not implemented in this study, these responses suggest that participants envision apps such as this one as comprehensive asthma management platforms. Other suggestions included integrating the app with other health management tools for an aggregated view of health data that could be shared with health care providers, facilitating a better understanding of respiratory health trends over time.

### Exacerbation Characteristics

Table 7 shows the relationship between PEF zones and asthma well-being responses from sessions with valid RRVB scores in the analysis cohort. The table includes 10.71% (224/2091) of sessions classified as exacerbations (yellow-red PEF zones and not good–bad well-being), averaging 3.1 events per participant or 1.0 per participant per month. In addition, 61.45% (1285/2091) of the sessions were recorded under the green PEF zone and feeling good category, indicating normal function conditions. The remaining 27.83% (582/2091) of the sessions were classified as mild respiratory events. It should be noted that the actual prevalence of exacerbations may differ from these numbers as use of the study app was self-directed by participants.

**Table 7:** Combined relative peak expiratory flow (PEF) and asthma well-being responses from the analysis cohort (N=2091).

PEF zones (percentage of the personal best)	Asthma well-being response, n (%)			
	Feeling good	Not feeling good	Feeling bad	Total
Green (≥80%)	1285 (61.5)	227 (10.9)	8 (0.4)	1520 (72.7)
Yellow (50%-79%)	340 (16.3)	181 (8.7)	25 (1.2)	546 (26.1)
Red (<50%)	7 (0.3)	14 (0.7)	4 (0.2)	25 (1.2)
Total	1632 (78)	422 (20.2)	37 (1.8)	2091 (100)

### Additional Asthma Characteristics

#### Number of Symptoms and Severity

The relationship between overall symptom severity and the number of reported symptom types is illustrated in Table S3 in Multimedia Appendix 1. As the number of symptoms increased, there was a notable shift toward higher severities, with moderate to severe symptoms occurring in <20% (377/2065, 18.26%) of all study sessions overall. However, among the small subset of sessions with 4 or 5 symptoms, 93% (87/94) were rated as moderate or severe, indicating a strong association between symptom count and perceived severity. Predominantly, in 77.43% (1599/2065) of the sessions, participants reported no symptoms or only 1 symptom type, highlighting the generally mild nature of the respiratory events captured in the study and the appropriateness of the RRVB for real-world, nonacute monitoring.

#### Symptom Types

To investigate the prevalence of different asthma symptoms, symptom types were categorized by respiratory state—normal function, mild event, and exacerbation—and normalized by the number of sessions in each state, yielding the prevalence percentage for each symptom type, as shown in Table S4 in Multimedia Appendix 1. The average number of symptom types reported increased progressively from 0.55 at normal function to 1.2 during mild events and 2.4 during exacerbations. Cough was the most prevalent symptom even for sessions during normal function (304/1285, 23.65%), suggesting limited specificity as an isolated marker of exacerbation. Following in order of decreasing prevalence were shortness of breath, notably prevalent for sessions during exacerbations (163/224, 72.8%) and emerging as the most discriminative symptom; wheezing; chest tightness or pain; and trouble sleeping. Participants were trained to select multiple symptoms as applicable, although the app interface did not explicitly state *select all that apply*, which may have led to underreporting in some sessions. These data illustrate that exacerbations are marked by significant objective and subjective respiratory impairment and multiple concurrent symptoms.

#### Asthma Triggers

The analysis of asthma triggers revealed varying prevalence across the 3 respiratory states, as shown in Table S5 in Multimedia Appendix 1. Allergies were the most identified trigger, affecting 52.7% (118/224) of exacerbation sessions and 38.45% (804/2091) of study sessions overall, followed by pollution at 24.15% (505/2091) and stress at 18.84% (394/2091). *All other* triggers, including tobacco smoke, disinfectants, and unspecified causes, also rose sharply during exacerbations (177/224, 79.0%) compared to normal function (564/1285 43.89%). No triggers were identified in 31.56% (660/2091) of the sessions. The average number of triggers reported per session increased from 1.5 at normal function to 1.6 during mild events and 2.2 during exacerbations, highlighting increased trigger exposure or reporting in more severe respiratory states. Stress was an exception, decreasing in prevalence during exacerbations compared to normal function and mild events, possibly reflecting its role as a background rather than acute factor. It should be noted that normal function sessions reported one or more triggers in 59.92% (770/1285) of the sessions, with an average of 1.5, indicating that trigger exposure alone is not reliably indicative of symptom severity.

#### Rescue Medication Use

Analysis of rescue inhaler use provides insight into participants’ asthma management strategies and their effectiveness. As indicated in Table S6 in Multimedia Appendix 1, the most common pattern was no reported use of rescue inhalers on the study day, observed in 44.32% (913/2060) of sessions. Among these, 80.5% (735/913) were classified as normal function states with few exacerbations noted, suggesting effective ongoing management or absence of significant symptoms and reinforcing *no use* as a marker of good respiratory status.

Participants reported using rescue inhalers earlier in the day to relieve symptoms in 41.12% (847/2060) of the sessions, indicating reactive management. Of these sessions, 46.0% (390/847) were at normal function, suggesting that inhaler use was effective approximately half the time. Conversely, 18.1% (153/847) of uses were during exacerbations, reflecting a lack of efficacy in these cases.

Proactive management was evident in 10.29% (212/2060) of sessions in which participants planned to use a rescue inhaler shortly after their session. Just over half (123/212, 58.0%) of the sessions were classified as mild events or exacerbations, indicating a delayed reactive response to worsening symptoms. The remaining 42.0% (89/212) of the session were classified as normal function, suggesting anticipatory use to manage well-controlled conditions or minor symptoms.

In addition, 4.27% (88/2060) of sessions involved inhaler use before exercise, with 86% (76/88) of these uses occurring at normal function or during mild events, indicating that proactive use before physical activity was mostly effective.

### RRVB Dataset and Quality Control Outcomes

#### RRVB Dataset Characteristics

Participants completed a total of 2922 app sessions, averaging 34.8 sessions per participant. This equates to approximately 11.6 sessions per month or 2.7 sessions per week. Voice sample quality control through the elicitation check and 2-sample scoring determined that 71.6% (2093/2922) of these sessions provided valid RRVB results (described in the following sections), which were used in the RRVB analyses.

The RRVB scores were normalized by subtracting individual reference values. This process included 68% (57/84) of the participants (normalized cohort), with 32% (27/84) excluded from the analysis cohort for having <5 normal function sessions. The median number (IQR=20) of normal function samples per participant was 17, allowing for robust RRVB reference estimation. The threshold for differentiating low and high risk on the normalized RRVB scores was calculated to be 3.7, representing the mean difference between the 70th and 50th percentiles of the reference scores across participants.

#### Elicitation Check

An elicitation check was applied to each collected voice sample in real time, allowing for immediate rerecording if the initial attempt failed quality control. Of the 5652 initial samples, 750 (13.3%) failed the elicitation check, leading to 744 second attempts. Of these 744 second attempts, 263 (35.3%) failed the elicitation check, necessitating a third attempt for 260 samples, of which 116 (44.6%) failed the elicitation check again. This progression shows the effectiveness of multiple attempts as only 2.05% (116/5652) of cases failed all 3 times. In total, 90.34% (2640/2922) of the total number of sessions successfully produced 2 RRVB scores that passed the elicitation check.

#### Two-Sample Scoring

Each app session was designed to collect 2 good-quality voice samples for comparative analysis and scoring. Voice samples that passed elicitation check quality control underwent further evaluation using a 2-sample scoring algorithm, which checked consistency between 2 RRVB scores obtained in the same app session. As a result, 79.28% (2093/2640) of the sessions with 2 scores that both passed the elicitation check were confirmed as consistent, and the 2 RRVB scores in these sessions were averaged to provide a final score. The 2 quality control mechanisms, the elicitation check and 2-sample scoring, provided increased confidence in the validity of RRVB scores and gave outputs for 71.62% (2093/2922) of the total app sessions. Voice samples from the remaining 28.37% (829/2922) of the sessions were not scored or used for RRVB analyses.

The relative number of sessions with 1 or 2 failed elicitation check samples or inconsistent 2-sample scoring results were similar for all respiratory states (normal function, mild events, and exacerbations). Consequently, neither quality control method nor the combination of both altered the distribution of respiratory states in the quality-controlled data compared to the original data.

## Discussion

### Vocal Biomarkers as an Asthma Monitoring Tool

This study advances the application of vocal biomarker technology in asthma management via a smartphone-based tool in a prospective real-world study. RRVB scores, derived from 6-second held [ɑ] vowel (as in *father*) voice recordings, provided immediate respiratory function feedback and significantly correlated with exacerbations, including in demographic and clinical subgroups. Exacerbation risk was more than twice as large in the high versus low RRVB score range, and this RR increased to approximately 3.5 using normalized RRVB scores. The tool also identified milder respiratory events, although with lower performance. Many participants remained engaged over the 3-month study, reporting favorable opinions and benefits. Unlike previous studies that have explored speech-based vocal biomarkers and home-based monitoring in limited capacities [11], our research uniquely integrated an existing vocal biomarker algorithm that provided real-time feedback to participants in real-world settings over an extended duration. This operationalization of voice-based exacerbation monitoring using everyday technology without requiring additional devices demonstrates the feasibility of patient self-monitoring and enhances asthma management accessibility. Building on the initial RRVB validation in cross-sectional studies [15], these results extend the RRVB’s use cases to longitudinal monitoring.

The RRVB normalization method successfully improved exacerbation detection and reduced the subgroup differences observed using raw RRVB scores. This suggests that the normalized scores are more accurate in capturing true respiratory dynamics versus the possible confounding impact of demographic, clinical, or device-related factors and highlights the suitability of longitudinal monitoring using the RRVB approach.

Participant feedback provided valuable insights into the usability and effectiveness of the RRVB tool. High satisfaction levels in the feedback questionnaire indicated that the core objectives—to assess the feasibility and user experience of a novel voice-based tool for asthma monitoring—were met positively. Participants appreciated the convenience; accessibility; and potential for regular, in-home monitoring provided by the app. This aligned with the study’s aim to offer a noninvasive and user-friendly approach to asthma management, with many preferring the vocal approach over a peak flow meter, likely due to its ease of use, consistent with studies comparing peak flow to symptom monitoring [2].

Both raw and normalized RRVB scores showed limited utility in reflecting self-reported asthma control assessed via ACT despite their effectiveness in stratifying momentary respiratory states. This discrepancy may stem from the static averaging method used, which fails to capture dynamic fluctuations in asthma symptoms. In previous studies, manual counting of cough frequency overnight or over 24-hour periods was demonstrated to correlate with asthma control, but this required extended recording intervals and manual approaches [8,9]. Future research could explore more advanced statistical modeling to analyze RRVB time-series data, potentially providing better insights into asthma control by accounting for temporal patterns and symptom variability.

The difference in the measures used—momentary exacerbations versus ACT—may also contribute to this discrepancy rather than indicating a failure of alignment. RRVB effectively differentiated exacerbations from normal function as it captured immediate respiratory function based on PEF and self-reported well-being, which participants reported in real time. In contrast, the ACT relies on a 4-week recall period, reflecting broader symptom trends and overall control rather than immediate states. This recall period can introduce bias that is not reflected in RRVB scores [20]. Clinicians often rely on standard measures such as the ACT or informal self-reports of past symptoms, lacking access to historical data that were captured in real time. This is an important distinction and highlights the complementary nature of longitudinal RRVB and ACT scores. Used together, they may offer a more complete picture of asthma status than either alone.

In addition to the RRVB scores, tools such as this one can capture a wealth of other structured data—such as symptoms, severity, triggers, and rescue inhaler use—that could further enrich the clinician’s insight into patient condition and behavior over time. The symptom and medication use patterns observed in this study mirror well-established asthma phenotypes and reinforce the ecological validity of the RRVB signal in real-world conditions. These continuous, patient-reported data offer a more detailed and reliable historical record compared to patients’ verbal recollection of symptoms and behaviors during clinical visits. It also enables linkage between momentary physiological signals (such as the RRVB) and behavioral indicators of disease management (such as rescue medication use), supporting more context-aware monitoring. The RRVB could fill a critical gap among self-perceived wellness (which can be delayed or imprecise), environmental exposures (which are frequent but not always predictive), and behavior (which may lag behind symptom onset). Such longitudinal, multilayered data open up avenues for artificial intelligence–driven analysis to identify key patterns, enabling more personalized and proactive patient care.

### Participant Engagement Factors

Achieving and sustaining high engagement is essential for health apps but often challenging. In this study, a promising 58% (49/84) of participants had medium to high engagement. These participants used the app on average 53 times over 3 months, 94% (46/49) used the app at least once in month 3, and 78% (36/49) remained consistently engaged (≥8 uses). These metrics compare favorably to those of many app-based studies, which often report sharp early drop-off and limited sustained use [21,22]. For example, Pratap et al [21] found a median retention of just 5.5 days across large-scale studies, and Amagai et al [22] noted that only approximately one-third of reviewed studies maintained majority engagement for their full duration. Our definition of high engagement (≥8 uses per month and activity into month 3) aligns with top-quartile patterns identified in these reviews and highlights the feasibility of RRVB use in sustained, real-world contexts.

Subgroup analysis (Table S1 in Multimedia Appendix 1) indicated lower engagement for male participants and those aged <30 years. In addition, there was a trend of lower engagement for those with mild asthma and Black or African American participants. These factors are consistent with reported asthma medication adherence issues, indicating potentially common underlying causes [23-25]. However, the 58% (49/84) of participants who were consistently engaged compare favorably to 14% to 16% of patients with *satisfactory* medication adherence in a 15-location pharmacy database study [23], highlighting a potential opportunity for the RRVB tool to encourage more proactive asthma management behaviors.

Comorbidities associated with higher engagement included COPD, diabetes, nonseasonal allergies, reflux disease, sleep apnea, skin conditions, and obesity. In contrast, participants with hypertension, seasonal allergies, and sinus conditions had similar engagement rates to those without these conditions. These engagement differences may reflect the chronic nature of conditions such as COPD, diabetes, and sleep apnea and their direct impact on respiratory function, potentially increasing the perceived need for continuous asthma monitoring. In contrast, conditions such as hypertension and seasonal allergies, which are often more episodic or managed via stable long-term treatments, may not drive the same level of engagement. In addition, mental health conditions such as depression and anxiety, which were also linked to higher engagement, may amplify health-related concerns, prompting more frequent use of monitoring tools. While this study was not designed to definitively determine these drivers of engagement, the results suggest that individuals with more complex or chronic comorbidities may be more motivated to actively manage their asthma through digital health tools.

### Limitations

This study’s selective participant group limits the generalizability of the results. While diverse, the participants may not reflect the broader population of patients with asthma, and the longitudinal nature may bias outcomes toward consistently engaged participants. All participants were required to have access to a smartphone and complete app-based sessions, which may exclude individuals with limited digital access or technological literacy, including older adults or those from more digitally disconnected segments of the population.

Despite these limitations, the RRVB tool’s demonstrated generalizability in 2 previous studies [15] and again on this new cohort represents a level of robustness rarely shown in the validation of machine learning–based tools. In particular, the RRVB tool demonstrated strong engagement and performance among Hispanic participants (Table S1 in Multimedia Appendix 1), suggesting its potential utility in traditionally underserved communities. This finding compares favorably to reported equity challenges in digital intervention effectiveness across diverse populations [26] and suggests that the RRVB can serve a broad range of patients when designed and deployed with accessibility in mind.

This study used PEF in combination with symptoms to define asthma state (normal function, mild event, or exacerbation) rather than as an outcome variable for respiratory function. While PEF is a convenient and commonly used tool in asthma management, it is effort dependent and can vary based on the patient’s performance during testing. In addition, PEF is not the gold standard for assessing airflow limitation, which may affect the accuracy of the respiratory state classification in our analysis. As a result, variations in PEF performance could lead to misclassification of asthma states, potentially impacting the RRVB comparison to true respiratory function.

While our study advances the field in important ways, including its prospective design, longer study duration, and real-world ecological implementation, some limitations remain. Compared to previous studies with smaller cohorts or retrospective analyses [11], our work demonstrates improved scale and automation. However, subgroup analyses (eg, by age, sex, or engagement levels) were limited in statistical power. In addition, while we used predefined symptom and PEF-based labels to reduce bias in state classification, further validation in broader and more diverse populations is warranted. Finally, while the RRVB tool successfully identified exacerbations and respiratory states in real time, it did not directly trigger clinical or self-management interventions. Participants were instructed not to use the RRVB scores for asthma decision-making during the study, and no automated alerts or care pathways were linked to RRVB outputs. As such, the tool currently functions as a monitoring aid rather than a stand-alone intervention system. Future integration of the RRVB into broader asthma management platforms with tailored feedback, alerts, or clinical decision support could improve its impact on timely intervention.

Because the RRVB tool was intentionally tested as a stand-alone application to assess its signal fidelity in real-world conditions, engagement and perceived utility might have been limited. Without integration into a comprehensive asthma management system, participants lacked additional functionalities that could enhance effectiveness, such as personalized asthma education or medication tracking. This limited context likely affected use patterns and participants’ assessment of the tool’s utility. Indeed, feedback survey responses indicated a need for more comprehensive guidance on score interpretation and actionable insights such as advice on using rescue medication or contacting health care providers. The feedback suggests that integrating the RRVB within broader asthma management platforms could enhance user engagement and provide valuable guidance. Linking RRVB results to tailored asthma educational content, as explored in ongoing studies integrating the RRVB into the ASTHMAXcel app [27,28], was also suggested by participants.

Feedback was obtained from 55% (46/84) of the enrollment cohort, primarily from more engaged participants. This may skew satisfaction and usability ratings toward a more positive evaluation, potentially overestimating the tool’s effectiveness. That said, satisfaction levels varied minimally by engagement level (separate analysis; data not shown).

### Opportunities for Impact

Existing digital asthma management platforms offer functionalities such as symptom tracking, medication management, peak flow and spirometry tracking, personalized asthma action plans, and access to educational resources [29]. Integrating advanced technologies such as the RRVB would represent a significant advancement in noninvasive, frequent monitoring of respiratory health. As digital platforms evolve, vocal biomarker technology is expected to offer a more proactive, personalized, and comprehensive approach to asthma management.

The RRVB’s ability to detect changes in respiratory status holds promise for improving asthma care by enabling a level of vigilance in disease management that is scalable, user-centric, and far beyond current capabilities. Frequent use of the RRVB could enhance patients’ perception of airflow limitation and medication adherence, as demonstrated with interventions using PEF prediction and feedback [30,31]. This suggests that the RRVB could help enhance patients’ awareness and management of their condition facilitated by the high engagement and positive reception demonstrated in this study. As a momentary signal that complements traditional tools such as the ACT, the RRVB could serve as a real-time input to future care models—flagging early changes in respiratory status that might prompt clinician review, self-management action, or digital triage. Practically, this could mean reduced frequency and severity of asthma attacks, better day-to-day symptom management, and potentially fewer urgent or emergency care visits and hospitalizations.

### Conclusions

This study evaluated the real-world utility of an RRVB for detecting asthma exacerbations using smartphone-based voice recordings in participant home settings. Through a 3-month prospective cohort design with daily app-based data collection, RRVB scores were shown to effectively stratify respiratory states and predict exacerbations with statistically significant RRs. Engagement was sustained in most participants, and user feedback indicated strong usability despite some noted areas for improvement. These results support the RRVB as a promising tool for continuous asthma monitoring and lay the foundation for future integration into more comprehensive self-management systems, including a potential role in proactive health tracking or patient-clinician communication.

## Appendix

Multimedia Appendix 1 Additional data tables S1-S6 (subgroup analysis, exit survey responses, participant-reported asthma symptom characteristics including number, type, severity, triggers and rescue medication use).

## Abbreviations

ACT: Asthma Control Test
COPD: chronic obstructive pulmonary disease
PEF: peak expiratory flow
RR: risk ratio
RRVB: respiratory-responsive vocal biomarker

## Data Availability

The datasets generated or analyzed during this study are not publicly available due to internal funding and proprietary considerations but are available from the corresponding author on reasonable request and subject to the execution of a standard data use agreement.
