# Supplementary material for: Respiratory-Responsive Vocal Biomarker for Asthma Exacerbation Monitoring: Prospective Cohort Study
Source: J Med Internet Res. 2025 Sep 23;27:e68741. doi: 10.2196/68741 (PMC12504899; doi:10.2196/68741)
Supplement: Multimedia Appendix 1 [file jmir_v27i1e68741_app1.docx]

This is a Multimedia Appendix to a full manuscript published in the J Med Internet Res. For full copyright and citation information see <http://dx.doi.org/10.2196/68741>

**Table S1.** Participant count, engagement (sessions), exacerbation prevalence (exacerbations / total session count) and RR for exacerbations (as in manuscript Table 4) by selected subgrouping variables to investigate differences within the study. Overall cohort results provided for reference in top row.

|  | | **Enrollment cohort** | | **Analysis cohort** | | **Normalized cohort** |
| --- | --- | --- | --- | --- | --- | --- |
|  | **Subgroup** | **Participants** count | **Session count** mean ± s.e.m. | **Exacerbation prevalence** | **Exacerbation RR** Raw RRVB | **Exacerbation RR** Normalized RRVB |
|  | **All** | 84 | 34.8 ± 2.8 | 10.7% | 2.15 (2.70-4.73, p<.001) | 3.57 (2.70-4.73, p<.001) |
| **Gender** | **Female** | 68 | 36.9 ± 3.2 | 10.8% | 1.79 (1.33-2.41, p=<.001) | 3.31 (2.44-4.49, p<.001) |
|  | **Male** | 16 | 25.9 ± 5.2 | 10.2% | 7.25 (2.63-20.00, p=<.001) | 5.34 (2.59-10.99, p<.001) |
| **Age range** | **18-30** | 23 | 27.0 ± 4.5 | 5.3% | 4.32 (1.73-10.79, p=0.002) | 1.80 (0.60-5.43, p=0.30) |
|  | **31-45** | 30 | 42.2 ± 4.1 | 10.2% | 2.94 (1.85-4.66, p<.001) | 3.76 (2.35-6.04, p<.001) |
|  | **45+** | 31 | 33.4 ± 5.5 | 13.9% | 1.00 (0.69-1.46, p=0.99) | 3.80 (2.65-5.45, p<.001) |
| **Ethnicity** | **Hispanic** | 49 | 33.1 ± 3.7 | 11.4% | 1.55 (1.10-2.20, p=0.013) | 4.96 (3.41-7.21, p<.001) |
|  | **Not Hispanic** | 35 | 37.1 ± 4.4 | 9.8% | 3.35 (2.06-5.44, p<.001) | 2.23 (1.44-3.46, p=<.001) |
| **Race** | **Black or African American** (1) | 34 | 30.2 ± 4.4 | 11.0% | 2.25 (1.37-3.70, p=0.001) | 2.65 (1.73-4.07, p<.001) |
|  | **All other races** | 50 | 37.9 ± 3.7 | 10.5% | 2.09 (1.48-2.96, p<.001) | 4.42 (3.03-6.46, p<.001) |
| **Asthma severity** | **Mild** (intermittent + persistent) | 36 | 35.1 ± 4.0 | 10.1% | 1.54 (1.04-2.28, p=0.03) | 3.42 (2.29-5.11, p<.001) |
|  | **Moderate** | 28 | 34.4 ± 4.9 | 9.0% | 3.91 (1.96-7.77, p=<.001) | 5.01 (2.41-10.43, p<.001) |
|  | **Severe** | 20 | 34.8 ± 7.0 | 13.7% | 2.12 (1.24-3.64, p=0.006) | 3.43 (2.18-5.40, p<.001) |
| **Asthma control** (2) | **Well controlled** | 16 | 44.1 ± 6.1 | 0.8% | 4.64 (0.49-44.27, p=0.18) | 1.88 (0.27-13.23, p=0.53) |
|  | **Not well controlled** | 16 | 38.0 ± 6.8 | 4.3% | 2.02 (0.60-6.78, p=0.25) | 6.78 (2.29-20.11, p=<.001) |
|  | **Poorly controlled** | 42 | 35.8 ± 3.9 | 18.9% | 1.83 (1.40-2.41, p<.001) | 3.21 (2.44-4.23, p<.001) |
|  | **No ACT** | 10 | 10.6 ± 5.1 | 1.6% | 0.18 (0.01-3.55, p=0.26) | 1.43 (0.06-32.65, p=0.82) |
| **Number of co-morbidities** | **0-1** | 17 | 27.1 ± 5.5 | 14.1% | 3.13 (1.38-7.06, p=.006) | 5.94 (2.66-13.31, p<.001) |
|  | **2-3** | 35 | 30.6 ± 4.6 | 6.6% | 2.26 (1.26-4.06, p=.006) | 2.16 (1.24-3.74, p=.006) |
|  | **4+** | 32 | 43.4 ± 6.1 | 13.0% | 1.86 (1.31-2.65, p<.001) | 3.69 (2.55-5.324, p<.001) |
| **Mental health condition diagnosis** | **None** | 45 | 31.1 ± 3.6 | 8.6% | 3.61 (2.17-6.00, p<.001) | 3.06 (1.89-4.95, p<.001) |
|  | **Any** | 39 | 39.1 ± 4.4 | 12.8% | 1.50 (1.06-2.11, p=0.02) | 3.59 (2.54-5.07, p<.001) |
| **Phone type** | **iPhone** | 53 | 36.1 ± 3.1 | 11.2% | 3.87 (2.62-5.71, p<.001) | 4.25 (2.95-6.11, p<.001) |
|  | **Samsung** | 26 | 32.6 ± 6.2 | 7.0% | 2.75 (1.11-6.82, p=.03) | 2.46 (1.37-4.37, p=.002) |
|  | **Other** | 5 | 32.8 ± 16.1 | 28.9% | 1.61 (0.68-3.80, p=.28) | 2.08 (1.11-3.92, p=.02) |

(1) Includes only participants indicating no other races beside Black or African American; (2) Based on average ACT per participant

**Table S2. Participant responses to the exit survey on usability and perceptions of the RRVB-based smartphone app for asthma monitoring (N=46). Participants rated agreement with each statement using a 5-point Likert scale. Free-text feedback was summarized thematically in the manuscript.**

| **Survey question** | **Completely agree** | **Somewhat agree** | **Neither agree nor disagree** | **Somewhat disagree** | **Completely disagree** |
| --- | --- | --- | --- | --- | --- |
| I like being able to check my asthma using a short voice recording on my smartphone | 30 | 11 | 3 | 2 | 0 |
| It was easy to record my voice and get respiratory scores in the app | 33 | 10 | 1 | 2 | 0 |
| I understand what the respiratory scores calculated from my voice recording mean | 24 | 8 | 4 | 6 | 4 |
| The respiratory scores calculated from my voice recording are helpful to me in understanding how I'm doing with my asthma | 26 | 10 | 4 | 4 | 2 |
| Overall, recording my voice on my smartphone is a better way to check my asthma than using a peak flow meter | 21 | 12 | 7 | 4 | 2 |
| If it was available to me, I would continue using an app similar to the one in the study to check my asthma in the future | 30 | 10 | 5 | 1 | 0 |
| The best thing about checking my asthma by recording my voice on my smartphone is: | Free text, summarized in the manuscript | | | | |
| The worst thing about checking my asthma by recording my voice on my smartphone is: |  |  |  |  |  |
| One thing that would make an app like this more useful for people with asthma is: |  |  |  |  |  |

**Table S3.** Reported number of symptoms (cough, shortness of breath, wheezing, chest tightness or pain, trouble sleeping) versus overall symptom severity from all study sessions with valid RRVB scores (counts and percent). Severity responses were missing from 34 study sessions and are omitted here.

| **Counts** (percent) | **Number of symptoms** | | | | | | |
| --- | --- | --- | --- | --- | --- | --- | --- |
| **Overall symptom severity** | **0** | **1** | **2** | **3** | **4** | **5** | **Total** |
| **N/A** | 778 (37.7) | 83 (4.0) | 12 (0.6) | 4 (0.2) | 0 | 0 | 877 (42.5) |
| **Mild** | 136 (6.6) | 453 (21.9) | 175 (8.5) | 40 (1.9) | 5 (0.2) | 2 (0.1) | 811 (39.3) |
| **Moderate** | 14 (0.7) | 135 (6.5) | 80 (3.9) | 42 (2.0) | 27 (1.3) | 6 (0.3) | 304 (14.7) |
| **Severe** | 0 | 0 | 5 (0.2) | 14 (0.7) | 17 (0.8) | 37 (1.8) | 73 (3.5) |
| **Total** | 928 (44.9) | 671 (32.5) | 272 (13.2) | 100 (4.8) | 49 (2.4) | 45 (2.2) | 2,065 |

**Table S4.** Likelihood of reporting symptom types by respiratory state (baseline, mild event, exacerbation) from all study sessions with valid RRVB scores. Calculated by adding up symptom type reports by respiratory state and dividing by total number of each respiratory state.

|  | **Likelihood of reporting symptoms by event category** (percent) | | | | | | **Avg. symptoms/ state** (count) |
| --- | --- | --- | --- | --- | --- | --- | --- |
| **Respiratory state** | **None** | **Cough** | **Shortness of breath** | **Wheezing** | **Chest tightness or pain** | **Trouble sleeping** |  |
| **Exacerbation** | 4 | 51 | 73 | 41 | 39 | 36 | 2.4 |
| **Mild** | 30 | 30 | 29 | 26 | 20 | 15 | 1.2 |
| **Baseline** | 58 | 24 | 10 | 7 | 7 | 8 | 0.55 |
| **Total** | **44** | **28** | **22** | **16** | **14** | **13** | **0.93** |

**Table S5.** Likelihood (percent) of reporting asthma trigger types by respiratory state (baseline, mild event, exacerbation) from all study sessions with valid RRVB scores. The “all other” category includes: tobacco, disinfectant, other (not specified), and unknown triggers.

|  | **Likelihood of reporting symptoms by event category** (percent) | | | | | | | **Avg. triggers/ state** (count) |
| --- | --- | --- | --- | --- | --- | --- | --- | --- |
| **Respiratory state** | **None** | **Allergies** | **Pollution** | **Stress** | **Pets** | **Exercise** | **All other** |  |
| **Exacerbation** | 2 | 53 | 33 | 13 | 24 | 17 | 79 | 2.2 |
| **Mild** | 24 | 38 | 24 | 21 | 16 | 12 | 50 | 1.6 |
| **Baseline** | 40 | 36 | 23 | 19 | 14 | 13 | 44 | 1.5 |
| **Total** | **32** | **38** | **24** | **19** | **16** | **13** | **49** | **1.6** |

**Table S6.** Frequency of reported rescue medication use, and relative occurrence of each respiratory state (baseline, mild event, exacerbation) as function of rescue medication use from all study sessions with valid RRVB scores. Medication use options sum to 100% across the top row; respiratory state relative occurrence sums to 100% across columns.

|  | **Rescue medication use response options** | | | |
| --- | --- | --- | --- | --- |
|  | **Plan to use after app session** | **Haven’t used today** | **Yes, used it earlier prior to exercise** | **Yes, used it earlier to relieve symptoms** |
| **Frequency of response option** | **10%** | **44%** | **4.3%** | **41%** |
| **Exacerbation** | 20% | 1.5% | 14% | 18% |
| **Mild** | 38% | 18% | 30% | 36% |
| **Baseline** | 42% | 81% | 57% | 46% |
